# Geometric models to explore mechanisms of dynamic shape change in skeletal muscle

**DOI:** 10.1098/rsos.172371

**Published:** 2018-05-16

**Authors:** Taylor J. M. Dick, James M. Wakeling

**Affiliations:** 1 School of Biomedical Sciences, University of Queensland, St Lucia, Australia; 2 Department of Biomedical Physiology and Kinesiology, Simon Fraser University, Burnaby, Canada

**Keywords:** force–velocity, gastrocnemius, gearing, Hill-type, pennation, ultrasound

## Abstract

Skeletal muscle bulges when it contracts. These three-dimensional (3D) dynamic shape changes play an important role in muscle performance by altering the range of fascicle velocities over which a muscle operates. However traditional muscle models are one-dimensional (1D) and cannot fully explain *in vivo* shape changes. In this study we compared medial gastrocnemius behaviour during human cycling (fascicle length changes and rotations) predicted by a traditional 1D Hill-type model and by models that incorporate two-dimensional (2D) and 3D geometric constraints to *in vivo* measurements from B-mode ultrasound during a range of mechanical conditions ranging from 14 to 44 N m and 80 to 140 r.p.m. We found that a 1D model predicted fascicle lengths and pennation angles similar to a 2D model that allowed the aponeurosis to stretch, and to a 3D model that allowed for aponeurosis stretch and variable shape changes to occur. This suggests that if the intent of a model is to predict fascicle behaviour alone, then the traditional 1D Hill-type model may be sufficient. Yet, we also caution that 1D models are limited in their ability to infer the mechanisms by which shape changes influence muscle mechanics. To elucidate the mechanisms governing muscle shape change, future efforts should aim to develop imaging techniques able to characterize whole muscle 3D geometry *in vivo* during active contractions.

## 1. Introduction

Observing the bulging biceps of a body builder or the wiry calves of a marathon runner indicates that skeletal muscle undergoes changes in geometry when it contracts. These three-dimensional (3D) shape changes emerge from muscles' ability to generate forces in multiple directions during a contraction. In fact, cross-bridges that form between the actin and myosin filaments act to pull them past each other in the longitudinal direction [1], but also develop forces to squeeze these myofilaments together in a radial direction [2]. Previous studies have shown that dynamic shape changes alter the force output of muscle by enhancing the range of lengths and velocities over which individual fibres operate and thus is likely a critical aspect of a muscle's mechanical performance [3–5]. Declines in locomotor performance with ageing and disease may be due to the limited ability for muscle to undergo changes in geometry in these conditions [6,7]. However, the mechanisms underlying *in vivo* shape changes in both healthy and pathological muscle are not fully understood.

Dynamic shape change is linked to the hierarchical organization of skeletal muscle and is influenced by the mechanical interactions of active and passive tissues spanning from the level of the sarcomere to the level of the whole muscle. During active shortening, actin–myosin cross bridges develop forces that cause fibres to shorten in length and compress in cross-sectional area [2]. Yet to remain (near) isovolumetric, forces develop in the opposite direction, causing fibres to bulge in girth [8] and thereby requiring muscle fibres to rotate to steeper angles. The magnitude and direction of fibre expansions and rotations are additionally affected by surrounding connective tissues that wrap around muscle fibres and span outer surfaces of the whole muscle belly (i.e. aponeurosis). Our previous work suggests that *in vivo* shape changes in the pennate human plantar flexor muscles are tightly linked to the internal constraints placed on an individual's muscle fibres—and more heavily influenced by force rather than velocity [5]. Similar to Azizi and colleagues [3] we found that at low force levels, shape changes allow for large amounts of fibre rotation, leading to a greater uncoupling of fibre velocity from whole muscle velocity (gearing: the ratio of muscle belly shortening velocity to muscle fibre shortening velocity) when compared to higher force levels. Despite the potential influence of muscle shape change on mechanical output, traditional Hill-type models—such as those used within whole body musculoskeletal simulations (e.g. [9])—greatly simplify (or even neglect) the dynamic shape changes that occur within contracting muscle.

In the traditional Hill-type model, the force generated by a muscle fibre is a function of its activation level, active and passive force–length properties, and force–velocity properties of the contracting fibres. These fibre forces can be scaled to represent whole muscle forces, with the addition of a geometrical representation, which is the pennation angle. In this formulation length and pennation angle are not related to each other; however within a contracting muscle, length and pennation angle are intimately linked (e.g. [5,10,11]). In order to account for this, an additional modelling constraint that relates length and pennation angle is added to the Hill-type model [12–14], and this is an assumption of constant thickness. Keeping the muscle thickness constant results in only one degree-of-freedom between length and pennation angle and so for convenience we describe this as a one-dimensional (1D) model. However, imaging studies have demonstrated that a muscle's thickness (distance between aponeuroses) as well as its pennation can change dramatically *in vivo* [4,10,15–18] and that the bulging of pennate muscles during contraction could have implications for the prediction of *in vivo* force. For example, when fibres rotate, increasing both pennation and muscle thickness during low-force high-velocity contractions, the fibres shorten slower than the muscle belly [3–5] in a process known as gearing. In effect, the force–velocity characteristics of the fibres are decoupled from those of the muscle belly. While 1D models do allow fibre velocity to be different from muscle--tendon unit (MTU) or muscle belly velocity, they assume thickness remains constant and thus do not allow gearing to increase if the muscle bulges during contraction. Muscle models that neglect this variable ‘gearing’ therefore may underestimate *in vivo* force.

Randhawa & Wakeling [11] have previously shown that it is possible to change the relationship between length and pennation angle using a series of geometric models that satisfy 1D, 2D or 3D constraints. Briefly, the two-dimensional (2D) model incorporates one additional parameter, aponeurosis compliance, which allows the muscle to bulge in thickness while maintaining a constant area. The 3D model can bulge in thickness and in width with the addition of aponeurosis compliance and a shape factor, which specifies the ratio of thickness and width shape change, while maintaining a constant volume. Although Randhawa and Wakeling determined that the 1D model predicts pennation angle with high accuracy (*r*^2^ = 0.99) during steady maximal contractions, gearing is known to vary with submaximal activations [5] and during time-varying cyclical contractions [4], implying that there is no unique pennation angle for a given fibre length as is predicted by a 1D model. Hence it is important for muscle models that incorporate 1D, 2D or 3D constraints to be tested under a greater range of time-varying submaximal conditions. Unfortunately, there remains relatively little data available on the dynamic 3D geometry changes of actively contracting human muscle that would allow detailed 3D models to be validated. In recent years, diffusion tensor imaging (DTI) has emerged as a potentially promising tool to quantify 3D *in vivo* muscle shape changes [19,20] and is likely the best source for comparison against predictions of geometric muscle models. However to date, DTI is limited to either static resting or passive lengthening conditions due to the considerable scanning time; therefore B-mode ultrasound imaging remains the best approach for model comparisons.

The purpose of this study was to compare predictions of muscle fascicle behaviour (length changes and rotations) between the traditional Hill-type model with 1D geometry to predictions from models that incorporate 2D and 3D geometric constraints. We compared time-varying estimates of fascicle length, pennation angle and gearing predicted by 1D, 2D and 3D Hill-type models to *in vivo* measurements from B-mode ultrasound during a range of submaximal dynamic contractions. We hypothesized that the 3D model would provide more accurate predictions of fascicle length, pennation angle, and gearing as compared to the 1D and 2D models. Previously, Randhawa & Wakeling [11] have shown that the 3D model performs slightly better than the 1D and 2D models, and this is the basis for our hypothesis. However, in this study we aim to determine how the 3D model compares against the 1D and 2D models during submaximal dynamic contractions, which are most relevant to locomotion.

## 2. Material and methods

### 2.1. Acquisition of experimental data

**Figure 1.**
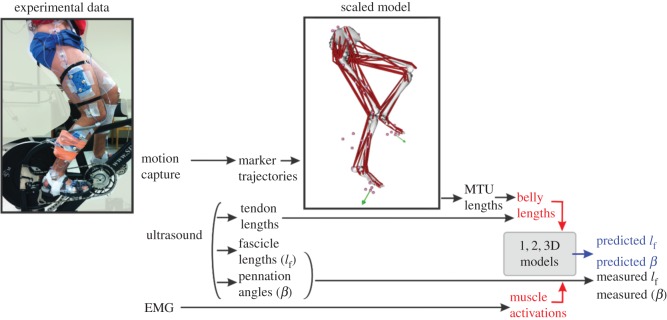
Approach for testing 1D, 2D and 3D muscle models using experimental data collected during human cycling. During the experimental protocol, subjects pedalled on a stationary bike while we measured 3D marker trajectories, tendon lengths, fascicle lengths, pennation angles and surface EMG. A musculoskeletal model was scaled to each subject and used to calculate the MG muscle–tendon unit (MTU) lengths during cycling. The difference between the time-varying MTU lengths, from the scaled musculoskeletal model, and the tendon lengths, from tracked ultrasound images, was used to determine muscle belly lengths during cycling. Geometric muscle models, of varying dimensions, were driven with the experimentally determined belly lengths and muscle activations, determined from EMG. Model predictions of fascicle length and pennation angle were compared against the experimentally measured values. We examined whether models' predictions changed when we allowed the aponeurosis to stretch (2D model), or when we allowed the aponeurosis to stretch and the fascicles to bulge in thickness and width during shortening (3D model). Refer to table 2 for definitions of all symbols used within models.

We collected a comprehensive set of kinematic, kinetic, electromyographic (EMG) and ultrasound data from 10 competitive cyclists (6 males, 4 females, age 33 ± 6 years; figure 1). Full details of the experimental protocol are described in Dick *et al*. [21] and Dick & Wakeling [5] and thus a brief overview is provided here. Subjects pedalled on a stationary cycle ergometer (Indoor Trainer; SRM, Julich, Germany) at cadences between 80 and 140 r.p.m. and crank torques between 14 N m and 44 N m while we recorded ultrasound image sequences of the medial gastrocnemius (MG) muscle belly and muscle–tendon junction (MTJ), the 3D trajectories of 32 LED markers, reaction forces normal and radial to the crank, and surface EMG patterns from 10 muscles. All subjects gave informed consent, and protocols were approved by the Institutional Review Boards at both Simon Fraser University and Harvard University.

Subjects pedalled at nine combinations of cadence and crank torque: 80 r.p.m. at 14 N m, 80 r.p.m. at 26 N m, 80 r.p.m. at 35 N m, 80 r.p.m. at 44 N m, 100 r.p.m. at 13 N m, 100 r.p.m. at 26 N m, 120 r.p.m. at 13 N m, 120 r.p.m. at 24 N m and 140 r.p.m. at 13 N m, corresponding to crank powers of 115, 220, 290, 370, 135, 270, 165, 300 and 200 W, respectively. Each trial lasted 15 s and each block of conditions was presented in a random order and repeated twice to enable imaging of both the MG muscle belly and the MG MTJ.

A B-mode ultrasound probe (7 MHz, 60 mm field of view, Echoblaster; Telemed, Vilnius, Lithuania) was secured over either the MG muscle belly or the MG MTJ on the right limb using a stretchy adhesive bandage, and an ultrasound gel pad (Parker Laboratories, NJ, USA) was placed between the probe and skin to enhance image quality and allow for muscle bulging. Ultrasound images were recorded at 40 Hz (and subsequently combined for 10 consecutive cycles), and prior calibration [22] determined the position of the ultrasound scanning plane relative to a triad of optical markers on the probe so that 2D ultrasound images of the MTJ could be projected into the 3D laboratory coordinate system. Ultrasound data collected from the muscle belly and the MTJ were synchronized using the 3D coordinates of LED markers placed on the right pedal.

On the left limb, bipolar Ag/AgCl surface EMG electrodes (10 mm diameter, 21 mm spacing, Norotrode; Myotronics) were placed over the mid-belly of the MG and nine other muscles (not reported here). EMG signals were sampled at 2000 Hz (bandwidth 10–500 Hz; Biovision, Wehrheim, Germany), amplified (gain 1000). ‘Maximum effort’ sprint trials (both high power and cadence) were collected at the beginning and end of each test session in an effort to elicit maximum muscle activity, and we used these data as a reference when normalizing the muscles' EMG intensities (e.g. [23]). Optical motion capture (Certus Optotrak; NDI, Waterloo, ON, Canada) sampling at 100 Hz was used to track the 3D locations of 32 LED markers placed bilaterally on the lower extremities, the pedals and the ultrasound probe. Static calibration trials were collected to scale a musculoskeletal model to each subject (OpenSim v3.3) [9].

### 2.2. Analysis of experimental data

Ultrasound images from the MG muscle belly and MTJ were manually digitized (ImageJ; National Institutes of Health, Bethesda, MD) to estimate time-varying fascicle lengths, pennation angles and tendon lengths during cycling (figure 1). Eight points in each ultrasound image of the muscle belly were digitized: three points on each of the superficial and deep aponeuroses and two points on the same representative muscle fascicle in the mid-belly region of each image. The aponeuroses were defined based on a linear fit of the three points. Pennation angle was calculated in each image as the mean of the two angles made by the fascicle with the deep and superficial aponeuroses. Fascicle lengths and pennation angles from 10 complete crank revolutions were fitted with 2-harmonic Fourier series for each subject at each condition.

Ultrasound images from the distal MTJ were manually digitized to estimate time-varying Achilles tendon (AT) lengths during pedalling. AT length was determined by the distance from the 3D coordinates of the AT insertion on the calcaneus to the digitized MG MTJ projected into 3D laboratory coordinate space. Time-varying MG MTU lengths were determined using a subject-specific scaled musculoskeletal model together with the experimental LED marker data via inverse kinematics (OpenSim v3.3) [9,24] (figure 1). Time-varying MG muscle belly lengths were calculated as the difference between MTU length from the scaled musculoskeletal model and tendon length from the tracked ultrasound images.

Instantaneous muscle belly velocity and fascicle velocity were calculated as the first time-derivative of the changes in belly length and fascicle length, respectively. Muscle belly gearing was calculated as the ratio of instantaneous muscle belly velocity to muscle fascicle velocity at the time of maximum belly shortening velocity. Maximum belly shortening velocity and maximum fascicle shortening velocity occurred within 8° of each other.

MG EMG intensity was calculated across a 10 to 450 Hz frequency band using an EMG-specific wavelet analysis [25] and was normalized by the maximum intensity detected during the ‘maximum effort’ reference trials. Since muscle force is linearly related to the EMG amplitude and not its power [26], we used the square-root of the normalized EMG intensity as a measure of muscle excitation ($\hat{e}(t)$). To determine normalized muscle activation $\hat{a}(t)\;$ from normalized muscle excitation $\hat{e}(t)$ we used the first-order differential equation [12] that is commonly used for modelling human muscle:

2.1 $$\frac{\text{d}}{\text{d}t}(\hat{a})+\left[\frac{1}{\tau_{\mathrm{act}}}(\gamma +[1-\gamma ]\hat{e}(t))\right]\;\hat{a}(t)=\left(\frac{1}{\tau_{\mathrm{act}}}\right)\;\hat{e}(t)$$

with a value of 35 ms for the activation time constant *τ*_act_ and 0.6 for the ratio *γ* of the activation to deactivation time constants.

### 2.3. Multidimensional muscle models

**Figure 2.**
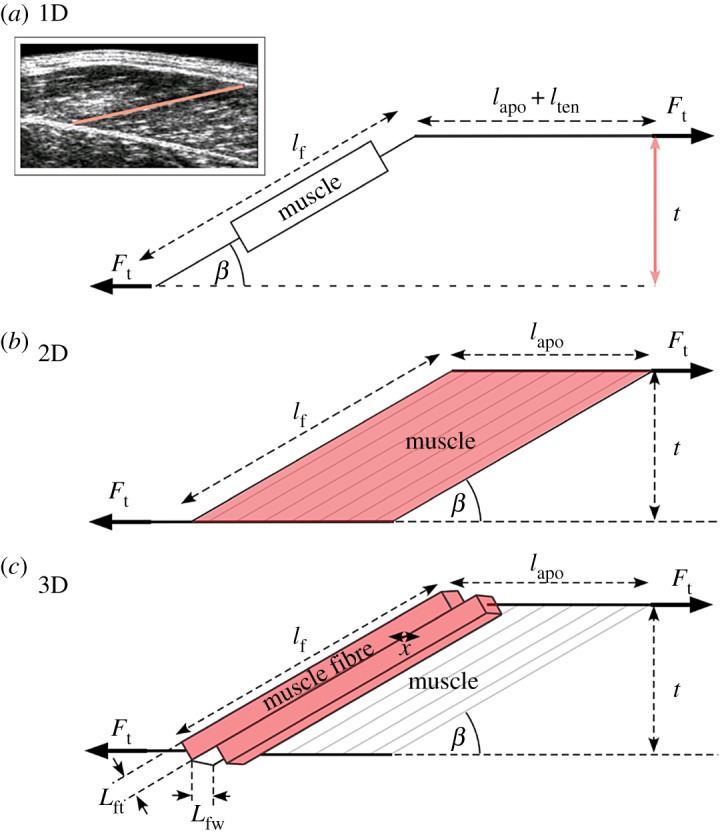
Schematic representation of 1D, 2D and 3D muscle models styled after Randhawa & Wakeling [11]. The 1D model assumes thickness (*t*) remains constant (*a*). The 2D model assumes that the area of the muscle remains constant (*b*). The 3D model assumes that the volume of the muscle fibre remains constant (*c*). (*l*_f_—fascicle length; *l*_ten_—tendon length; *l*_apo_—aponeurosis length; *L*_ft_—fascicle thickness; *L*_fw_—fascicle width; *β*—pennation angle; *t*—thickness; *F*_t_—tendon force). Top panel in (*a*) shows an ultrasound image of the medial gastrocnemius muscle belly with a fascicle highlighted in red.

The three geometric models differ in their geometric constraints, but all derive their fibre forces from an underlying Hill-type model that is driven with muscle fibre activation, length and velocity (figure 2). These geometric models are consistent with those derived in Randhawa & Wakeling [11]. However in contrast to this previous study where force estimated from dynamometer torque was used to drive geometric models, here we estimate force using the Hill-type formulation and thus are able to better account for the muscle's physiological state. Specifically we used muscle activations determined from surface EMG and belly lengths *l*_b_ determined from the difference between MTU length, derived from a scaled musculoskeletal model, and tendon length derived from B-mode ultrasound, to drive models. Fascicle length *l*_f_ and pennation *β* were predicted by the Hill-type models that incorporated 1D, 2D and 3D geometric constraints. The models are presented here using fascicle length *l*_f_ which can be calculated as

2.2 $$l_{f}=\frac{l_{\mathrm{apo}}-l_{b}}{\cos\beta },$$

where *l*_f_ is equal to the difference between aponeurosis length *l*_apo_ and belly length *l*_b_, divided by the cosine of pennation angle *β*.

### 2.4. One-dimensional model

The 1D model is based on the traditional Hill-type model (e.g. [11,27]) where a contractile element is in series with an elastic element (figure 2). The contractile element represents the muscle fascicle (*l*_f_), arranged at a pennation angle *β* relative to the force-generating axis of the muscle. In the 1D model, the thickness remains constant, where

2.3 $$l_{f,i}\;\sin\;\beta_{i}=l_{f,c}\;\sin\;\beta_{c}$$

such that current pennation angle *β*_c_ and fascicle length $l_{f,c}$ are dependent on initial pennation angle *β*_i_ and initial fascicle length $l_{f,i}$ and there is only one pennation angle for a given fascicle length. Initial conditions for *l*_b_, $\hat{a}(t)$, as well as *l*_f_ and *β* for each trial were determined from the experimental data at 270° of the crank cycle before the muscle was active and were specific to each subject and condition.

### 2.5. Two-dimensional model

The 2D model considers the muscle as a panel with pennate fascicles that originate and insert on two parallel aponeuroses in line with the external tendon [28] (figure 2*b*). In the 2D model, the area remains constant, where

2.4 $$l_{f,i}\;\sin\;\beta_{i}l_{\mathrm{apo},i}=l_{f,c}\;\sin\;\beta_{c}l_{\mathrm{apo},c}.$$

The aponeurosis has a compliance *C*_apo_, which is the strain that would occur at maximum isometric force. Thus the aponeurosis can stretch as a function of force, such that if *C*_apo_ = 0.15 it would stretch 15% of its initial length at maximum isometric force. The amount of stretch depends on the fascicle force $\left({\hat{F}}_{f}\right)$ in the direction of the muscle's line of action. Fascicle force was calculated using a Hill-type model:

2.5 $${\hat{F}}_{f}=\hat{a}(t){\hat{F}}_{a}(l_{f}){\hat{F}}_{a}(v)+{\hat{F}}_{p}(l_{f}),$$

where ${\hat{F}}_{f}$ is a function of the time-varying activation $\hat{a}(t),$ the normalized active and passive force–length relationships ${\hat{F}}_{a}(l_{f})$ and ${\hat{F}}_{p}(l_{f})$, respectively, and the normalized force–velocity relationship ${\hat{F}}_{a}(v)$. The active and passive force–length curves, the maximum intrinsic speed, and the force–velocity relationship used in these models are provided in electronic supplementary material, file 1, and have been previously described in Dick *et al*. [21].

The predicted ${\hat{F}}_{f}$ can be used to determine how much the aponeurosis stretches:

2.6 $$\frac{l_{\mathrm{apo},c}}{l_{\mathrm{apo},i}}=1+C_{\mathrm{apo}}{\hat{F}}_{f}\;\cos\;\beta_{c}.$$

Combining equations (2.4) and (2.6) gives

2.7 $$\sin\;\beta_{i}=\frac{l_{f,c}}{l_{f,i}}\;\sin\;\beta_{c}(1+C_{\mathrm{apo}}{\hat{F}}_{f}\;\cos\;\beta_{c}).$$

### 2.6. Three-dimensional model

The 3D model considers that muscles can bulge not only in thickness but also in width, and this can be modelled using an additional shape factor *n*, which relates the bi-directional bulging of individual fascicles (in the thickness and width dimensions [11]) (figure 2*c*). If *n* = 0.5 then the fascicle bulges equally in width and in thickness. In the 3D model, the volume of fascicles (and thus the whole muscle) remains constant to satisfy

2.8 $$\frac{l_{f,c}}{l_{f,i}}\frac{l_{\mathrm{ft},c}}{l_{\mathrm{ft},i}}\frac{l_{\mathrm{fw},c}}{l_{\mathrm{fw},i}}=1,$$

where the product of the changes in the normalized dimensions along the fascicles' length *l*_f_, thickness *l*_ft_ and width *l*_fw_ equals 1. Decreases in fascicle length *l*_f_ must be matched by increases in the cross-sectional area of the fascicle, and these changes can occur in thickness and in width. Shape factor *n* relates the thickness and width-wise expansion of the fascicles:

2.9 $${\left(\frac{l_{\mathrm{ft},c}}{l_{\mathrm{ft},i}}\right)}^{n}={\left(\frac{l_{\mathrm{fw},c}}{l_{\mathrm{fw},i}}\right)}^{\left(1-n\right)}.$$

The normalized distance $\hat{x}$ between the fascicle centres in the longitudinal direction (figure 2*c*) is

2.10 $$\hat{x}=\frac{l_{\mathrm{ft},c}}{l_{\mathrm{ft},i}}\frac{\sin\;\beta_{i}}{\sin\;\beta_{c}}.$$

$\hat{x}$ is also the distance between the fascicle centres where they insert onto the aponeurosis, which is equal to the normalized length of the aponeurosis:

2.11 $$\hat{x}=\frac{l_{\mathrm{apo},c}}{l_{\mathrm{apo},i}}=1+C_{\mathrm{apo}}{\hat{F}}_{f}\;\cos\;\beta_{c},$$

where the normalized aponeurosis length $\left(l_{\mathrm{apo},c}\text{/}l_{\mathrm{apo},i}\right)$ depends on the aponeurosis compliance (*C*_apo_), normalized fascicle force (${\hat{F}}_{f}$) and current pennation angle (*β*_c_). Combining equations (2.9), (2.10) and (2.11) gives

2.12 $$\sin\;\beta_{i}={\left(\frac{l_{f,c}}{l_{f,i}}\right)}^{\left(1-n\right)}\;\sin\;\beta_{c}(1+C_{\mathrm{apo}}{\hat{F}}_{f}\;\cos\;\beta_{c}).$$

Note that equation (2.12) can also describe the bulging of a pennate muscle in 1D if *n* = 0 and *C*_apo_ = 0 (equation (2.3)) and in 2D if *n* = 0 and *C*_apo_ > 0 (equation (2.7)). The mechanical properties and length changes of the external tendon (figure 2*a*) were not included in any of the models.

### 2.7. Model comparisons

We used the 1D, 2D and 3D models and performed simulations by varying *C*_apo_ from 0 to 0.15 and *n* from −0.2 to 1. A value of *C*_apo_ between 0 and 0.15 indicates that the aponeurosis can stretch between 0% and 15% at maximum isometric force and a value of *n* between −0.2 to 1 corresponds to the changes fascicle dimensions presented in table 1. While the physiological range for *C*_apo_ has been measured in numerous studies with reported values typically between 1% and 10% [30–38], there remains less information on the physiological values for *n*. Randhawa & Wakeling [11] report optimized values of *n* = 0.025 for the human MG, which corresponds to a 0.25% increase in fascicle width accompanied by a 9.7% increase in fascicle thickness for 10% fascicle shortening. Whereas a finite-element modelling study reports values of 7.83% and 0.74% for the changes in fascicle width *l*_fw_ and fascicle thickness *l*_ft_, respectively, which corresponds to an approximate value of *n* = 0.91 [29]. Varying the values of *C*_apo_ and *n* beyond the physiological range reported in the literature allowed us to explore 1D, 2D and 3D models with varying levels of aponeurosis compliance and various combinations of fascicle shape change.

**Table 1.** Physiological representation of fascicle shape factor *n*. Values represent changes in fascicle width *l*_fw_ and fascicle thickness *l*_ft_ for a 10% shortening of the muscle fascicles. Negative values indicate a decrease.

*n*	Δ*l*_fw_ (%)	Δ*l*_ft_ (%)
−0.2	−2	12
0.025^a^	0.02	9.7
0	0	10
0.5	5	5
0.91^b^	7.83	0.74
1	10	0

^a^Value from Randhawa & Wakeling [11].

^b^Value based on results in Rahemi [29].

Models were driven with time-varying belly lengths *l*_b_, and activations $\hat{a}(t)$. Initial conditions were required for *l*_b_, $\hat{a}(t)$, as well as for *l*_f_ and *β* at each condition. These were determined from the experimental data during pedal upstroke before the muscle was active and were specific to each subject and condition. Models predicted time-varying fascicle lengths *l*_f_ and pennation angles *β*, and these were compared to the experimentally measured values. We calculated the models' predicted gearing by taking the first time-derivative of *l*_f_ to calculate predicted *v*_f_. We characterized differences between the models' predicted muscle parameters *l*_f_ and *β* and the *in vivo* ultrasound-based measures of *l*_f_ and *β* across the entire pedal cycle using two measures: the coefficient of determination *r*^2^ and the root mean square error (RMSE). We also compared the predicted gearing to the measured gearing, determined at the time of maximum belly shortening velocity, by calculating the absolute difference between the predicted and the measured ratio of belly velocity to fascicle velocity. The effect of model type (1D, 2D, 3D), experimental condition (crank torque and pedalling cadence) and subject (random factor) were tested using a general linear model ANOVA. Tukey *post hoc* comparisons were used to determine differences between the individual models. Differences were considered significant at the *p* *<* 0.05 level. Values are reported as mean ± s.e.

## 3. Results

**Figure 3.**
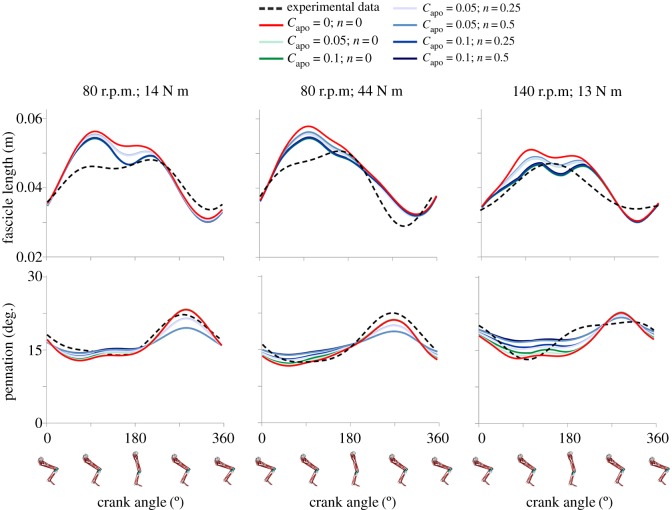
Comparison of experimentally measured and model predicted time-varying fascicle lengths and pennation angles. Data are shown for fascicle lengths (top row) and pennation angles (bottom row) measured using ultrasound (dashed black line) and predicted from the 1D (red line), 2D (green lines), and 3D (blue lines) Hill-type models varying the values of aponeurosis compliance *C*_apo_ and shape factor *n* used within each model. Data are shown for one representative subject pedalling at 80 r.p.m. at 14 N m (left panel), 80 r.p.m. at 44 N m (middle panel) and 140 r.p.m. at 13 N m (right panel).

The 1D, 2D and 3D models tested here were able to capture the general features of the ultrasound measurements of fascicle length and pennation angle (figure 3). However, when compared to the experimental data (black dashed line), all models overestimated fascicle excursions, overestimated fascicle rotations, and predicted peak fascicle length at an earlier time in the pedal cycle when compared to the experimental data. For all models, the time-varying patterns of predicted and measured fascicle lengths and pennation angles, as assessed by *r^2^* and RMSE, showed greater differences at high pedalling cadences as compared to low cadences (*p* < 0.05) (figure 3). The 1D model (red) predicted larger absolute changes in both fascicle length and pennation angle in comparison to the 2D (green) and 3D (blue) models (figure 3). The largest difference in model predictions occurred between the 1D model (red) and the most extreme 3D model (dark blue) where *C*_apo_ = 0.1 and *n* = 0.5 (figure 3). There were greater differences between the 1D and 2D model predictions and between the 1D and 3D model predictions at low load (crank torque) as compared to high load conditions (*p* *<* 0.05).

**Figure 4.**
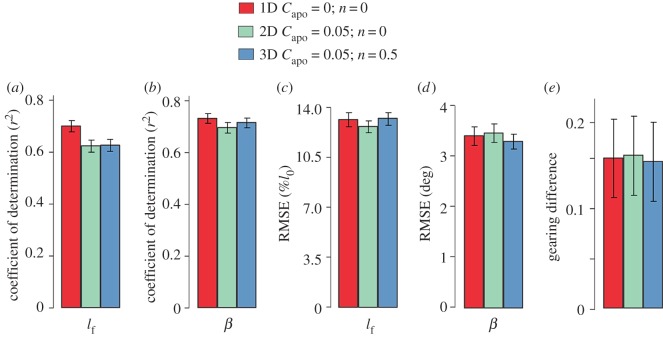
Model predictions for fascicle length, pennation angle and gearing showed minor differences between the 1D, 2D and 3D models*. r*^2^ and RMSE comparing the measured and predicted fascicle lengths (*a*,*c*), pennation angles (*b*,*d*) and gearing differences (*e*) for a selection of 1D, 2D and 3D models. Each set of bars represents the 1D (red), 2D (light green) and 3D (blue) model predictions as compared to the experimentally measured parameter. Each bar represents the average across subjects and pedalling conditions with standard error bars shown. RMSE for fascicle length was normalized to optimal fascicle length for each subject. Gearing differences were calculated as the absolute difference between measured gearing and the model-predicted gearing. Positive values indicate experimental gearing was greater than predicted gearing.

Surprisingly, there were minor differences in predicted fascicle geometry between the different models tested here. When comparing the predicted time-varying fascicle length and pennation angle across the pedal cycle for a 1D model, a 2D model with *C*_apo_ = 0.05, and a 3D model with *C*_apo_ = 0.05 and *n* = 0.5, there were no significant differences in the *r^2^* or RMSE between any of the models (figure 4). The 1D model predicted the measured changes in fascicle length with an *r^2^* of 0.70 ± 0.021 and the measured changes in pennation angle with an *r^2^* of 0.73 ± 0.019. The 2D and 3D models had *r^2^* values similar to those of the 1D model: 0.62 ± 0.024 and 0.63 ± 0.024 for fascicle length and 0.70 ± 0.019 and 0.72 ± 0.019 for pennation angle for the 2D and 3D models, respectively (figure 4*a*,*b*). RMSEs were similar for the three different models and ranged from 13.4 to 13.5% *l*_0_ and 3.28° to 3.39° for fascicle length and pennation angle, respectively (figure 4*c*,*d*). Gearing predictions between the 1D, 2D and 3D models were also similar, with differences ranging from 0.158 to 0.165 (figure 4*e*). The experimentally measured gearing was consistently greater than the predicted gearing for all 1D, 2D and 3D models tested here.

**Figure 5.**
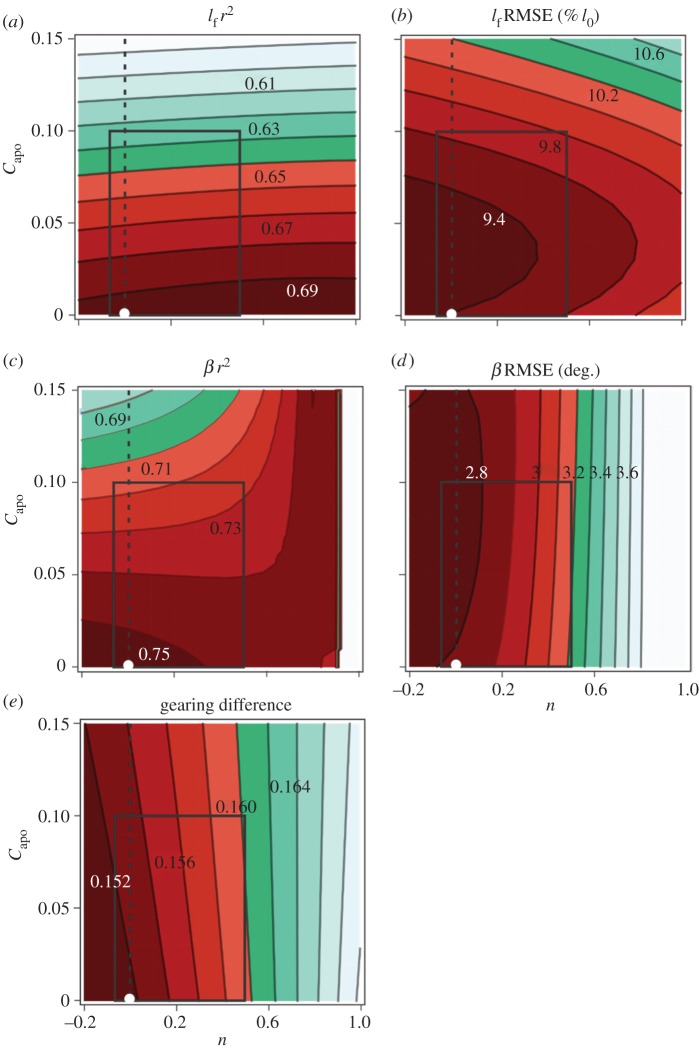
Contour plots for the *r*^2^ (*a*,*c*) and RMSE (*b*,*d*) comparing experimentally measured and model predicted fascicle length, pennation angle, and gearing differences (*e*) for the range of *C*_apo_ and *n* values tested in this study. Contour plots show the comparison between model predictions and experimental data when we varied aponeurosis compliance *C*_apo_ from 0 to 0.15 and fascicle shape factor *n* from −0.2 to 1.0. Darker colours indicate a better fit (higher *r^2^* or lower RMSE) between the experimental data and model predictions. RMSE for fascicle length was normalized to optimal fascicle length. Gearing differences were calculated as the difference between measured gearing and predicted gearing. Positive values indicate that experimental gearing was greater than predicted gearing, with lower values indicating a better fit between model and experimental values. On each of the contour plots, the white circle indicates the comparison between the experimental measurement and the 1D model prediction; the dashed line indicates the comparison for the range of 2D models where *C*_apo_ ranged from 0 to 0.15, and the box indicates the comparisons for 3D models within physiological range for *C*_apo_ and *n* reported in the literature.

When we altered the models’ aponeurosis compliance (*C*_apo_) and fascicle shape factor (*n*), the 1D, 2D and 3D models showed small differences in their ability to predict time-varying fascicle lengths and pennation angles during pedalling. *r^2^* varied from 0.61 to 0.69 and from 0.69 to 0.75 for fascicle length and pennation angle, respectively, when *C*_apo_ varied from 0 to 0.15 and *n* varied from −0.2 to 1 (figure 5*a*,*c*). RMSE varied from 9.4 to 10.6% *l*_0_ and from 2.8° to 3.6° for fascicle length and pennation angle, respectively, when *C*_apo_ varied from 0 to 0.15 and *n* varied from −0.2 to 1 (figure 5*b*,*d*). The 1D model generated similar predictions of fascicle length and pennation angle as compared to 2D model with low aponeurosis compliance (*C*_apo_) and 3D model with low *C*_apo_ and small values for fascicle shape factor (*n*). Overall, the absolute differences between the 1D, 2D, and 3D models within the range of *C*_apo_ and *n* values tested here were small: Δ*r*^2^ for fascicle length = 0.06; Δ*r*^2^ for pennation angle = 0.04, and ΔRMSE for fascicle length = 0.2% *l*_0_; ΔRMSE for pennation angle = 0.4°. The differences in predicted gearing and measured gearing between the 1D, 2D and 3D models were also small—they varied by 0.008 within the physiological space tested (figure 5*e*).

## 4. Discussion

In this study we used Hill-type models with 1D, 2D and 3D geometric constraints to test mechanisms of dynamic muscle shape change during submaximal contractions. The traditional 1D Hill-type model assumes a muscle cannot change in thickness and may therefore underestimate *in vivo* muscle force during contractions where force–velocity characteristics of the muscle fibres are uncoupled from those of the whole muscle due to changes in muscle thickness, pennation and gearing. In addition to the 1D model, we tested 2D and 3D models that spanned and exceeded the reported physiological values of aponeurosis strain (0%–15%; *C*_apo_ = 0–0.15) and the relative amounts of muscle bulging in thickness and width, defined as the fascicle shape factor (*n* = −0.02 to 1; table 1). Surprisingly, we found minor differences in the predictions of fascicle length, pennation angle and gearing between the 1D, 2D and 3D models. However, the differences between the three models' predictions were most pronounced at the lowest load condition—where changes in muscle geometry and gearing were greatest [5].

### 4.1. Multidimensional Hill-type models

All three models used in this study provided reasonable predictions for fascicle length and pennation angle from measurements of muscle belly length and activation, with *r*^2^ values greater than 0.62 and 0.70 for fascicle length and pennation angle, respectively. The *r*^2^ values in this study are substantially less than those reported in Randhawa & Wakeling [11], where *r*^2^ was greater than 0.96 for all three models. The differences in model performance between these two studies are likely related to differences in both the experimental conditions as well as the model formulations. First, Randhawa and Wakeling's measurements were taken during seated plantar flexion contractions at maximum activation under either constant speed or constant load conditions. They subsequently used measured fascicle length to predict pennation angle in all three models. In contrast, we took measurements during pedalling under time-varying submaximal activations at varying speeds and loads. During pedalling the gastrocnemii function in both ankle plantar flexion and knee flexion and the level of activation, length, and velocity vary throughout the pedal cycle [5,21,39]. Second, Randhawa and Wakeling did not implement the Hill-type formulation (equation (2.5)) within their models but rather estimated muscle force from the torque measured on the dynamometer and moment arms estimated from literature. In contrast, we estimated force using a Hill-type model driven with surface EMG derived activation and ultrasound-based muscle belly length estimates. Thirdly, our geometric models predict fascicle length and pennation angle given a set of initial inputs whereas Randhawa and Wakeling predict pennation angle given initial pennation angle and fascicle length throughout the entire movement cycle.

From a computational perspective, the fact that the 1D model is a specific scenario of the 3D model with *n* = 0 and *C*_apo_ = 0 may make this model formulation (equation (2.12)) superior because it encompasses all 1D, 2D and 3D situations. However, the 3D model incorporates additional parameters, *C*_apo_ and *n*, and although *C*_apo_ has been reported in numerous studies, there remains relatively little data on the value for *n*. Therefore, if the purpose of a model is for general utility, for example to predict muscle fascicle length and pennation angle from muscle belly length and activation within multi-joint dynamic musculoskeletal simulations, then consistent with Randhawa & Wakeling [11] our results would suggest that the simple 1D model (equation (2.3)) is likely sufficient. Based on the findings of our study, it is likely that during submaximal dynamic tasks *C*_apo_ is low (less than 2.5%) and *n* is low or negative. These values are similar to those optimized for the MG in Randhawa & Wakeling [11] (table 2). More specifically these results suggest that, under constant volume constraints, changes in fascicle girth during contraction predominantly occur in the thickness direction as compared to the width direction.

**Table 2.** Model parameters. Indices c = current and i = initial for fascicle and aponeurosis dimensions.

symbol	definition
*â*(*t*)	activation
*β*	pennation angle
*C*_apo_	aponeurosis compliance
*ê*(*t*)	excitation
${\hat{F}}_{a}(l_{f})$	normalized active force–length relationship
${\hat{F}}_{a}(v)$	normalized active force–velocity relationship
*F*_f_	fibre force
${\hat{F}}_{p}(l_{f})$	normalized passive force–length relationship
*l*_apo_	aponeurosis length
*l*_b_	muscle belly length
*l*_f_	fascicle length
*l*_ft_	fascicle thickness
*l*_fw_	fascicle width
*n*	fascicle shape factor
*t*	muscle thickness
*τ*_act_	activation time constant
*w*	muscle width
$\hat{x}$	normalized distance that fascicle cross-sections occupy along the muscle line of action
*γ*	ratio of the activation to deactivation time constants

### 4.2. Influence of aponeurosis properties on dynamic shape change

The 2D and 3D models implemented here allow us to understand the role of aponeurosis strain in dynamic muscle shape changes within a unipennate muscle. We found that differences in fascicle length and pennation angle predicted by a 1D model, where the aponeurosis was unable to stretch, compared to a 2D or 3D model with an aponeurosis compliance of 5% were small: less than 1% *l*_0_ for fascicle length and less than 1° for pennation angle. Although the presence of the aponeurosis substantially contributes to stress asymmetries that lead to dynamic muscle shape changes [29], our results suggest that the underlying effects of aponeurosis compliance alone on variable muscle shape change are potentially minor. This is further supported by results from the 2D model: when aponeurosis compliance varies from 0% to 15% we find minor differences in fascicle length (0.8% *l*_0_) and pennation angle (0.1°). This may not be surprising when considering that the influence of aponeurosis stretch on muscle shape changes is likely only as important as far as the aponeurosis can stretch. Under the highest activation levels measured here and assuming the aponeurosis stretches 6% from its initial length at maximum force (based on the average from literature values), we would predict that the aponeurosis stretches about 5 mm. This is about a quarter of the extent to which fascicles shorten during pedalling [5] and even less when compared to fascicle behaviour during other locomotor tasks such as walking and running [40,41]. When contracting muscles bulge, the aponeuroses not only strain longitudinally, but also transversely (width-wise) [4,29,42]. During active contractions, these biaxial strains have been shown to modulate longitudinal aponeurosis properties, whereby increases in aponeurosis width lead to increases in longitudinal stiffness (decrease in *C*_apo_). Although the models presented here did not explicitly test the influence of these biaxial properties, our results suggest that reducing aponeurosis compliance (equivalent to increasing stiffness) from 3% to 1% has relatively minor effects on fascicle behaviour and gearing. Further, transverse aponeurosis strains have been shown to plateau at approximately 20% of maximum isometric force [29], and thus these biaxial effects are likely more functionally relevant at low levels of force and during active lengthening contractions which are different mechanical conditions from those tested in the current study.

We must note that the aponeurosis may play a different role in muscle shape change depending on the nature of the experiments and the architectural complexity of the muscle. Here we used B-mode ultrasound to non-invasively look under the skin at dynamic muscle behaviour during human pedalling while previous studies on shape change have typically been performed on isolated *in situ* preparations of single muscles removed from an animal and placed on a muscle ergometer (e.g. [3,6]). Given that the human MG is associated with the neighbouring lateral gastrocnemius and the underlying soleus it is likely that shape changes, fascicle behaviour and force output are affected by the mechanical interactions with surrounding muscles and connective tissues [43–45] or bone. It is therefore possible that the influence of the aponeurosis on constraining shape changes is different between physiologically intact systems measured *in vivo* during time-varying motor tasks and single isolated muscles measured *in situ* during force and activation constrained contractions; however this remains largely untested. In addition, muscles with increasingly complex fascicle architectures have been shown to have inhomogeneous aponeurosis strains during active contractions [46], and it is possible that in these muscles, aponeurosis compliance may have a larger effect on muscle shape changes.

Aponeurosis sliding rather than aponeurosis stretch potentially plays a key role in modulating the variations in fascicle shortening velocity and gearing observed here. When pennate muscle shortens, the aponeuroses slide past each other considerably—this leads to fascicle shortening and changes in pennation angle [47]. As mentioned previously, the influence of the aponeurosis on the internal muscle geometry is likely only as important as far as the aponeurosis can stretch (up to 6%), which is less than the amount it slides (approximately 30% of total muscle belly length change; estimated from the values presented in table 2 and fig. 4 from [47]). Huijing & Woittiez [47] used a geometric model together with experimental data in the rat MG and found that the ability for muscle fibres to shorten and rotate during contraction is tightly linked to the considerable aponeurosis sliding (previously defined as tendon plate sliding). They concluded that this phenomenon allows fibres to undergo smaller length changes than that of the whole muscle belly (in a manner akin to gearing) and operate on more favourable positions of their force–length relationship. Similarly, Bolsterlee and colleagues [20] used DTI methods to estimate aponeurosis length change during passive lengthening contractions and found relatively minor, if not negligible, changes in aponeurosis length. Together, these results and ours may suggest that the traditional 1D model, where the aponeuroses are able to slide but cannot stretch, may be sufficient to predict fascicle behaviour and gearing.

### 4.3. Are constant volume muscle models appropriate?

The isovolumetric nature of skeletal muscle during contraction is assumed by nearly all muscle models (e.g. [12,21,48,49]), including those tested here. While constant volume has been demonstrated at the level of the whole muscle [50], muscle fibre [51] and the myofilament [52], there is additional evidence to suggest that fibre bundles [53] and whole muscles [54] are not isovolumetric during contraction. More recent DTI techniques, which are able to quantify the multiscale 3D architecture of whole human muscles *in vivo*, found a small decrease (1.6%) in MG volume during passive lengthening [20]. Although physiologically plausible, the authors note that this volume change may be an artefact of manual muscle segmentation errors. In the 3D model tested here, *n* is calculated under the assumption that the volume of muscle remains constant, where decreases in fascicle length must be matched by increases in the cross-sectional area of the fascicle (in some combination of thickness and width) (equation (2.8)). However future models could relax this constraint and allow volume to decrease during contraction, which is possible, for example through the loss of blood from intramuscular capillary networks. Although the models presented here have not explored this effect, small changes in muscle volume potentially lead to significant changes in *n*. This would result in variable muscle shape changes in thickness and width that are dependent on the amount of volume change. If true, it is plausible that constant volume models may not be appropriate to fully explain *in vivo* dynamic muscle shape changes. Indeed, the nature and importance of potential volume changes on the mechanical behaviour of skeletal muscle warrants further investigation.

### 4.4. Limitations

The results presented here must be considered within the context of the experimental and modelling limitations. There were some discrepancies between the temporal alignment of the experimentally determined fascicle lengths and those predicted by the different models (figure 3). The models were driven with muscle belly lengths, determined from the difference between MTU length estimated from a scaled musculoskeletal model, and tendon length determined from ultrasound measurements. Experimental fascicle lengths were determined from digitized ultrasound images of the MG muscle belly. It is likely that differences in the timing between tendon length changes and fascicle length changes contributed to this discrepancy between predicted and measured fascicle lengths. However, we chose this approach based on recent work that suggests caution when inferring changes in tendon length from ultrasound measurements of fascicle lengths alone [55]. The greater discrepancies at the higher cadences may be related to the digitizing errors of ultrasound images at higher cadences. Previously we have shown that Hill-type models of human muscle predict, on average, 54% of the force measured *in vivo* during submaximal dynamic tasks [21]. It is possible that some of the discrepancies between the models' predicted fascicle lengths, pennation angles, and gearing and those measured using ultrasound were a result of inaccuracies in the Hill-type models' force predictions rather than the geometric models themselves (equations (2.3), (2.7) and (2.12)). However, the errors for these Hill-type models of human muscle [21] are equivalent to *in vivo* models from animal studies where the inputs were acquired using invasive experimental techniques (e.g. [48]).

The experimental fascicle lengths and velocities determined from the ultrasound images assumed the fascicles are linear, following paths from the superficial to the deep aponeurosis within the scanning plane of the ultrasound probe. But muscle fascicles are in fact curvi-linear [35,56,57] which is suggested to be necessary to maintain mechanical stability within the muscle [58]. In addition, 3D fascicle rotations during dynamic contractions may cause fascicles to rotate and curve out of the 2D scanning plane [57]. However, 2D B-mode ultrasound measurements of pennate muscles report errors of less than 6% for fascicle length [35] and less than 1° for pennation angle [57] when treating fascicles as linear rather than curvi-linear structures during 2D dynamic scanning, and thus the effects of this assumption on our results are likely minor. Further, it is possible that external compressive forces due to the stretchy adhesive bandage used to secure the ultrasound probe may have influenced the ultrasound measurements. Our previous work has shown that a tubular compressive bandage leads to a 0.02 decrease in gearing [59]. To limit the effects of probe compression on our results we used strips, rather than tubes, of adhesive bandage and additionally a gel ultrasound pad to allow the muscle to bulge underneath the probe surface.

## 5. Conclusion

The ability for muscle to generate force to power movement is dependent on, among other physiological features, the lengths and velocities of its contracting fibres. When muscle shortens it undergoes 3D shape changes that affect its contractile behaviour and motor output [3,5,6]. Given that the ubiquitous Hill-type model is 1D and neglects dynamic shape changes, we aimed to test whether the addition of one or two dimensions could improve its predictive capacity. We found that a 1D Hill-type model predicted fascicle lengths and pennation angles similar to both 2D and 3D models that allowed either the aponeurosis to stretch (2D) or both the aponeurosis to stretch and variable muscle shape changes to occur (3D). This suggests that if the intent of a model is to predict the behaviour of muscle fascicles (length and pennation angle), then a traditional 1D Hill-type model may be sufficient. However we must note that 1D models do not allow us to infer the mechanisms by which shape changes influence muscle mechanics and in all of the models tested in this study, inconsistencies remained between the extent of the predicted changes in fascicle behaviour and the experimental measurements. Therefore, even the 3D model presented here may not be sufficient to study the geometric effects of skeletal muscle fascicles during contraction. The implementation of the 1D, 2D and 3D geometric models, together with the Hill-type formulation used in this study is novel. Particularly, models were driven by muscle activations and muscle belly lengths rather than fascicle lengths which allowed us to explore the effects of variable aponeurosis compliance and muscle shape changes on whole muscle behaviour within an intact MTU during submaximal dynamic tasks. Future work should aim to develop imaging techniques to better characterize the 3D shape changes of actively contracting muscle and the effects of these shape changes on *in vivo* muscle function. New imaging modalities have the potential to improve diagnosis and treatment for pathological conditions where shape changes may be restricted, for example due to elevated levels of intramuscular fat in diabetes mellitus [60] or due to increased extracellular matrix collagen cross-linking in muscular dystrophy [61].

## Supplementary Material

Supplementary file 1

## Data accessibility

Data are available from the Dryad Digital Repository: http://dx.doi.org/10.5061/dryad.8jm70 [62].
