# Supplementary material for: Geometric models to explore mechanisms of dynamic shape change in skeletal muscle
Source: R Soc Open Sci. 2018 May 16;5(5):172371. doi: 10.1098/rsos.172371 (PMC5990834; doi:10.1098/rsos.172371)
Supplement: Supplementary file 1 [file rsos172371supp1.docx]

***Hill-type parameters***

The normalized active force-length curve (Otten, 1987) was given by:

$$\hat{F_{a}}\left( l_{f} \right)=e^{-\left( \left( \frac{{\frac{l_{f}}{l_{0, f}}}^{0.6}-1}{0.3} \right)^{2.3} \right)}$$

The normalized passive force-length curve was given by:

$\hat{F}_{p}=2.64\left( \frac{l_{f}}{l_{0, f}} \right)^{2}-5.30\left( \frac{l_{f}}{l_{0, f}} \right)+2.66$ for $l_{f}>l_{0,f}$

$\hat{F}_{p}\left( l_{f} \right)=0$ for $l_{f}\leq l_{0, f}$

where $l_{f}$ is fascicle length and $l_{0, f}$is fascicle slack length. $l_{0, f}$was determined to be the length of the fascicle at tendon slack length. This passive force-length curve is similar to that provided by Millard and co-workers (2013) and is based on a combination of experimental data from chemically-skinned human gastrocnemius fibres (Gollapudi and Lin, 2009) and rabbit whole muscles (Winters et al., 2011).

The normalized force-velocity curve (Figure 3-1 (D)) was given by:

$\hat{F}_{a}\left( v \right)=\frac{1-\frac{\hat{v}}{v_{0}}}{1+\frac{\hat{v}}{v_{0}\alpha}}$ for $v\leq0$

$\hat{F}_{a}\left( v \right)=1.5-0.5\frac{1-\frac{\hat{v}}{v_{0}}}{1-\frac{7.56 \hat{v}}{v_{0}\alpha}}$ for $v>0$

**References:**

Gollapudi, S. K. and Lin, D. C. (2009). Experimental determination of sarcomere force–length relationship in type-I human skeletal muscle fibers. *J. Biomech.,* 42, 2011-2016.

Millard, M., Uchida, T., Seth, A. and Delp, S. L. (2013). Flexing computational muscle: modeling and simulation of musculotendon dynamics. *J. Biomed. Eng.,* 135, 021005.

Otten, E. (1987). A myocybernetic model of the jaw system of the rat. *J. Neurosci. Methods,* 21, 287-302.

Winters, T. M., Takahashi, M., Lieber, R. L., and Ward, S. R. (2011). Whole Muscle Length-Tension Relationships Are Accurately Modeled as Scaled Sarcomeres in Rabbit Hindlimb Muscles. *J. Biomech.,* 44, 109–115.
